# Genetics and prescription opioid use (GaPO): study design for consenting a cohort from an existing biobank to identify clinical and genetic factors influencing prescription opioid use and abuse

**DOI:** 10.1186/s12920-021-01100-z

**Published:** 2021-10-26

**Authors:** Vanessa Troiani, Richard C. Crist, Glenn A. Doyle, Thomas N. Ferraro, Donielle Beiler, Stephanie Ranck, Kortney McBryan, Margaret A. Jarvis, Jordan S. Barbour, John J. Han, Ryan J. Ness, Wade H. Berrettini, Janet D. Robishaw

**Affiliations:** 1 Geisinger Clinic, Geisinger, Danville, PA USA; 2 Department of Translational Data Science and Informatics, Geisinger, Danville, PA USA; 3 Neuroscience Institute, Geisinger, Danville, PA USA; 4 Department of Basic Sciences, Geisinger Commonwealth School of Medicine, Scranton, PA USA; 5 Center for Neurobiology and Behavior, Department of Psychiatry, University of Pennsylvania Perelman School of Medicine, Philadelphia, PA USA; 6 Department of Biomedical Sciences, Cooper Medical School of Rowan University, Camden, NJ USA; 7 Department of Pain Medicine, Geisinger Medical Center, Danville, PA USA; 8 Department of Biomedical Science, Schmidt College of Medicine of Florida Atlantic University, Boca Raton, FL USA

**Keywords:** Opioid use disorder (OUD), Genetics, Addiction, Substance misuse

## Abstract

**Background:**

Prescription opioids (POs) are commonly used to treat moderate to severe chronic pain in the health system setting. Although they improve quality of life for many patients, more work is needed to identify both the clinical and genetic factors that put certain individuals at high risk for developing opioid use disorder (OUD) following use of POs for pain relief. With a greater understanding of important risk factors, physicians will be better able to identify patients at highest risk for developing OUD for whom non-opioid alternative therapies and treatments should be considered.

**Methods:**

We are conducting a prospective observational study that aims to identify the clinical and genetic factors most stongly associated with OUD. The study design leverages an existing biobank that includes whole exome sequencing and array genotyping. The biobank is maintained within an integrated health system, allowing for the large-scale capture and integration of genetic and non-genetic data. Participants are enrolled into the health system biobank via informed consent and then into a second study that focuses on opioid medication use. Data capture includes validated self-report surveys measuring addiction severity, depression, anxiety, and nicotine use, as well as additional clinical, prescription, and brain imaging data extracted from electronic health records.

**Discussion:**

We will harness this multimodal data capture to establish meaningful patient phenotypes in order to understand the genetic and non-genetic contributions to OUD.

**Supplementary Information:**

The online version contains supplementary material available at 10.1186/s12920-021-01100-z.

## Background

Chronic pain is a major clinical problem in the United States, affecting 20% of adults, and it is one of the most common reasons that adults seek medical care [1]. Despite evidence indicating that high doses of prescription opioids (POs) are linked to an increased risk of opioid-related overdose death [2], many chronic pain patients are treated with opioids. In the United States, over 14,000 people died from overdoses involving POs in 2019 [3], but amidst the COVID-19 pandemic, nonfatal overdoses and overdose deaths have increased by 50–76% [4–7]. Misuse of these drugs was previously estimated to cost health insurance companies up to $72.5 billion a year [8]. Recent epidemiological estimates indicate that 2–5% of the United States population misuses POs [9]. At Geisinger, an integrated health system with > 2 million patients, where this study is based, over 300,000 patients have been treated with POs. A previous study on a small sample of patients being treated with POs at Geisinger indicates that 13.2–41.3% meet the criteria for moderate to severe opioid use disorder (OUD) [10]. The same study reports that depression, anxiety, illicit drug use, post-traumatic stress disorder, alcohol dependence, being under 65 years old, and patient-reported assessments of poor health are all associated with increased risk for OUD. In subsequent analyses of the Geisinger population, we find that patients treated with opioids for chronic non-progressive pain, who are enrolled in a contract-based medication management program, are much more likely to have characteristics of OUD determined via chart review, as well as comorbid conditions, such as depression and anxiety [11].

In addition to known clinical risk factors, there is also a strong genetic component to OUD and other substance use disorders [12]. Genome-wide association studies (GWAS) are a powerful approach that uses common allelic variants to identify genes and implicate specific biological pathways in certain disease states. GWAS can also be helpful in predicting the risk of certain diseases in subgroups of the population [13]. Previous GWAS analyses reveal certain genetic variation that may be linked to risk for developing OUD, including changes found in the coding regions of genes responsible for calcium and potassium channel function [12]. The largest GWAS of OUD to date uncovered only 1 statistically significant genetic variation, a single nucleotide polymorphism (SNP) in *OPRM1*, the gene encoding the mu-opioid receptor [14]. A major challenge for genetic studies of OUD is low statistical power due primarily to limited sample sizes and high phenotypic heterogeneity. Thus, one factor hindering discovery of genomic predictors of OUD is the ascertainment of well-characterized (i.e., deeply phenotyped) populations of individuals who are exposed to opioids without developing OUD, as well as those with confirmed OUD.

Longitudinal electronic health records (EHRs) are a digital version of a patient’s medical history and when harnessed for research, can provide real-world clinical data at population-scale. In health systems with a biobank, genotype data can be linked with existing clinical data to generate derived associations. EHR-derived phenotypes have advanced genomic discovery of major medical and psychiatric diseases [15–17], with the majority of derived phenotypes focusing on diagnostic codes, lab values, and medication data. One type of EHR data that has not been widely utilized for discovery is imaging data. For example, magnetic resonance images (MRIs) of the brain may further reveal important clinical insights about individuals using opioids. POs have been shown to cause structural changes in the brain after periods of use as brief as one month [18]. Substance abuse also causes recognizable structural brain changes; however, there are few studies that look specifically at structural changes subsequent to PO abuse. One study of brain MRIs in opioid abusers specifically excluded patients with a pain diagnosis [19]. Consequently, little is known about the structural and functional differences of chronic pain patients with and without OUD.

A more thorough understanding of the clinical and genetic risk factors for OUD is needed for genomic and neurobiological discovery, as well as to enable physicians to readily identify patients at high risk for OUD. Geisinger, with its large geographically stable population, research infrastructure, and status as an integrated health system, is ideal for a study that combines data from clinical, genomic, and patient-reported sources. Of primary relevance to studies of opioid use and abuse, Geisinger has a large chronic pain patient population, with over 30,000 patients currently receiving POs and over 300,000 with opioid exposure. Geisinger serves a primarily rural population (more than 12 Pennsylvania counties considered Appalachia) and has an existing biobank that holds specimens from nearly 200,000 patients with linked genetic sequence data, to date. The informed consent for the biobank allows for recontact of a highly engaged patient population: consent for the biobank protocol is > 85% [20]. Thus, the Geisinger environment provides a unique opportunity for patient recruitment into a longitudinal study of opioid use, abuse, and OUD.

In this prospective study, we plan to identify 20,000 patients who have been prescribed opioid analgesic medications at least twice in their lifetime (over 10,000 enrolled, to date). Patients are eligible to participate if they are between the ages 18–75 and of European ancestry. We determined to only include patients of European ancestry given the characteristics of Geisinger’s population (~ 96% white) and to improve power to detect genetic signal (see Methods and Discussion for plans to replicate in more diverse patient populations). Patients who are not already enrolled in the biobank protocol are invited to participate when consented for this study. Using this study design, we harness the rich diversity of data captured in EHRs and combine this with prospective self-reported questionnaire data from opioid-exposed and opioid-using patients, thereby establishing a cohort of genotyped and deeply phenotyped patients with a range of opioid use, misuse, dependence, and addiction.

## Methods/design

### Project overview

This is a prospective study utilizing standard questionnaires, chart review, genetic, and brain imaging data to determine possible clinical, genetic, and neuroanatomic traits that predispose an individual to opioid addiction.

### Recruitment

**Fig. 1:**
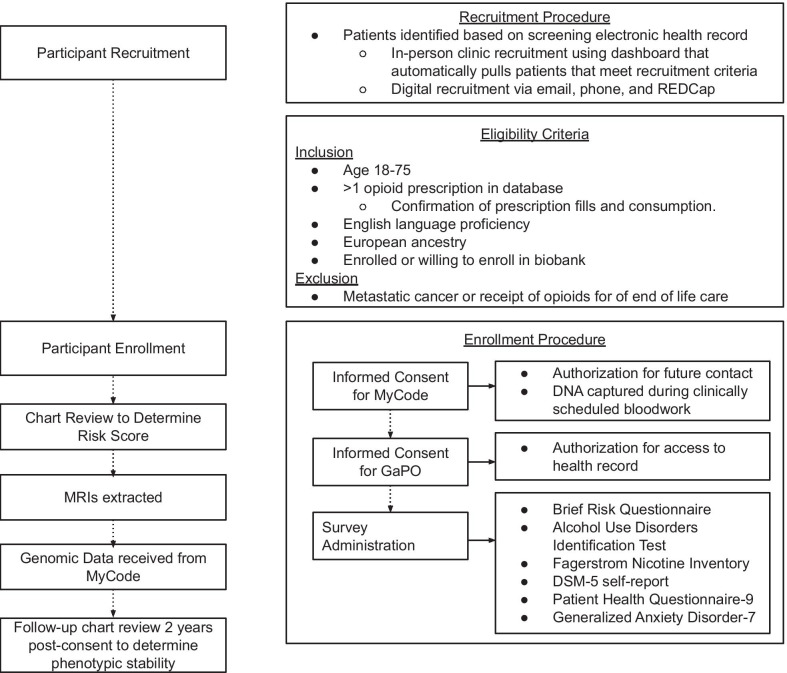
GaPO study visits, assessments, and data extraction flow chart

**Fig. 2:**
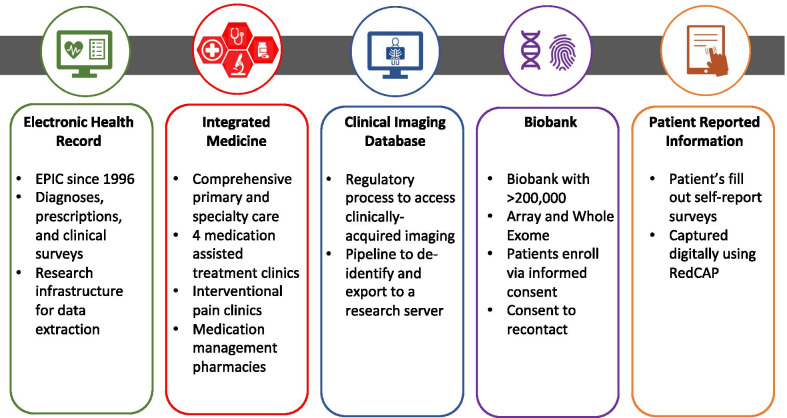
Relevant features of the Geisinger translational research infrastructure and study design of GaPO that allow for large scale genomic and phenotypic data capture

Patients are recruited from the health system using a multi-pronged recruitment strategy that harnesses the clinical-research infrastructure at Geisinger (See Figs. 1 and 2). A list of eligible patients that meet inclusion, but not exclusion, criteria (below and in Fig. 1) are identified by a Geisinger data broker. These eligibility lists are then aligned with specific Geisinger clinic schedules on a recruitment dashboard, allowing research assistants to approach prospective recruits to explain the study during their regularly scheduled clinic visits. If patients are already enrolled in the Geisinger biobank, MyCode, they are reminded of their participation and then interested patients are additionally consented into **GaPO**. For patients not already enrolled in the biobank, the research assistant explains MyCode and **GaPO** and consent is obtained for both studies. In addition to in-person clinic-based recruitment, patients are enrolled via a digital recruitment arm of the study. For digital recruitment, patients already enrolled in MyCode are sent information on the study via the patient portal or e-mail, and consent and study participation are achieved virtually using REDCap.

### Inclusion criteria

- Age 18–75
- Patients have received at least two opioid prescriptions over the course of their lifetimes
  - If < 3 prescriptions over a lifetime in the EHR prescription database, verbal confirmation from patient that the prescriptions were filled and multiple doses taken is required.
- Reads, writes, speaks, and understands English
- Self-identifies as European ancestry
- Is currently enrolled in the Geisinger biobank (MyCode) project, or consents to enroll in MyCode

### Exclusion criteria

- Has other severe debilitating disease which may interfere with assessment and response to opioid treatment for chronic pain (e.g., metastatic cancer, palliative end of life care)
- Not of European ancestry

We recruit patients from multiple clinics across Geisinger’s geographic service area that have a large proportion of patients with opioid prescriptions. We use the recruitment dashboards described above for each clinic type, which includes the interventional pain setting and pharmacy clinics assisting with complex medication management. In order to enrich our sample for patients that have a confirmed diagnosis of OUD, we also recruit from Geisinger’s Addiction Medicine Clinics [21], which serve patients undergoing outpatient treatment for substance use disorders.

### Study design

After providing informed consent to Geisinger’s biobank protocol (MyCode [20]) and **GaPO**, patients complete several questionnaires, taking ~ 20–30 min in total (or each?). The consent and questionnaires are completed using REDCap, either on an iPad at clinical appointments (with the help of a research assistant), or remotely, from a personal computer or other digital device. Questionnaires include: the Brief Risk Questionnaire (BRQ [18]), Fagerstrom Test for Nicotine Dependence (FTND [23]), Alcohol Use Disorder Identification Test (AUDIT [24]), Generalized Anxiety Disorder 7-Item (GAD-7 [25]), Patient Health Questionnaire-9 (PHQ-9 [26, 27]), and DSM Questions to determine OUD, amongst others (see Table 1).

**Table 1:** Self-reported surveys and questionnaires captured in patients enrolled in GaPO

Questionnaire name	Questionnaire purpose
Alcohol Use Disorders Identification Test (AUDIT)	The AUDIT is a 10-item, self report screener that assists in identifying excessive drinking and alcohol dependence [24]
Brief Risk Questionnaire© (BRQ)	The Brief Risk Questionnaire (BRQ) is a 12-item questionnaire that covers aberrant behavior related to medication use. Content of questions include personal and family history of substance abuse, legal issues and street drug use, past treatment, psychiatric history, and medication security [22]
Columbia-Suicide Severity Rating Scale (C-SSRS)	The C-SSRS is a questionnaire designed to assess suicide risk. We included three questions from the C-SSRS in order to determine whether a respondent was currently suicidal and required intervention. These questions were triggered if a respondent answered positively to the suicidal ideation question on the PHQ-9 survey (described in further detail below) [51]
Opioid Use Disorder Checklist	We adapted the DSM criteria for opioid use disorder to a self-report form in the current study. Patients completed an 14-item questionnaire and items are scored similarly to the interview version of the opioid use disorder checklist
Fagerstrom Test for Nicotine Dependence (FTND)	The Fagerstrom Test for Nicotine Dependence (FTND) is six-item survey that assesses the intensity of physical dependence on nicotine. Content includes questions about the quantity of cigarettes used per day, compulsion to use, and dependence [23]
First Use Questions	To better understand whether patients obtain opioids for the treatment of pain and via prescription as part of their first exposure, we ask two questions. (1) Was the treatment of pain the first reason you took opioids? (2) When using opioids, did you obtain the opioid as a prescription from a physician?
Generalized Anxiety Disorder (GAD-7)	The Generalized Anxiety Disorder scale (GAD-7) is a seven-item self-report measure that can aid in diagnosis and severity determinations of anxiety disorder [25]
Patient Health Questionnaire (PHQ-9)	The Patient Health Questionnaire (PHQ) is a nine-item diagnostic tool that can assist in making a diagnosis of depression and quantifies depression symptom severity [27]

After completion of the questionnaires, there is no further study-related patient contact. As part of the consent process, patients give permission to access their entire health record and give permission to link non-health record data (such as insurance claims, external prescription databases, etc.). Following enrollment, the study plan comprises several elements, including estimating an OUD risk score based on chart review criteria [11], extracting brain imaging using the clinical imaging database pipeline, genomic analyses, and assessment of phenotypic stability based on EHR review. More detailed analytic plans are described in the Data Analysis section, below.

## Data elements and survey instruments

### Genetic data

In 2007, Geisinger adopted an opt-in biobank protocol (MyCode, [20]), with most patients consented in-person by a research consenter in the context of regularly scheduled clinical appointments. To date, > 280,000 patients provided consent for the biobank, which captures DNA from blood samples obtained during standard clinical patient blood draw procedures. In 2014, Geisinger Health System and Regeneron Pharmaceuticals partnered to launch the DiscovEHR project [28]. The goal of this project is to use DNA samples from the MyCode participants to obtain genomic information from individuals and link it to their clinical data to better understand the genetic basis of diseases. Genetic data for the **GaPO** study is made available through MyCode/DiscovEHR, including whole exome sequencing (WES) and Human OmniExpressExome (HOEE) genotype data. To date, > 200,000 samples have been collected from consented patients and > 185,000 have been genotyped and sequenced from the MyCode/DiscovEHR cohort.

### Phenotypic data

In addition to DNA, patients [1] provide self-report data on survey questions and validated surveys and [2] give permission to access other existing and future data within their health record. Please see descriptions below, as well as Tables 1 and 2.

**Table 2:** Electronic health record data captured in patients enrolled in GaPO

Data variable	Variable description
ICD Codes	International Classification of Disease (ICD) codes are used in the Geisinger system to classify procedures, exams, and other patient encounters. They are used for diagnosis and billing purposes
Social History	Information relevant to social and behavioral determinants of health are captured within the Social History section of the electronic health record. Information captured includes patient smoking status and other substance use/abuse history, as well as the date this information was recorded within the health record
Demographics	Patient age, sex, race, gender, employment status, and address are available as part of the health record
Surveys Captured as Part of Clinical Care	Patients may be asked to complete surveys regarding aspects of their health at various appointments and visits while a patient at Geisinger. The name, description, version, and date of the survey are all stored in the medical record. Examples include the PHQ-2, a depression screener, which is captured on every patient during regularly scheduled primary care appointments. Other department-specific surveys include patient-reported pain scales captured during clinic visits for interventional pain treatment and Addiction Severity Index scores captured as part of substance abuse treatment
Prescription Data (from within and outside of Geisinger)	The medical record houses medication administration information, including drug name, dose, route and location of administration. Information is also available for medication dispensed outside of Geisinger via Surescripts^2^ (which is a health information network used by doctors, pharmacists, EHR companies, etc.). Surescripts data is normally pulled overnight or upon demand by a Geisinger provider. Upon request, it pulls back around 24 months of prior medication dispensed information
Procedures and laboratory results	There are procedure orders and procedure results available in the EHR, including surgical and non-surgical procedures. Information available includes the specific procedure that was ordered, description of the procedure order, and the type of procedure order. Procedure results also include lab results, whether the lab result was abnormal and type of abnormality, etc
Insurance claims	Which insurance company a patient has used for their Geisinger care is documented and accessible. Geisinger also has its own insurance company, Geisinger Health Plan (GHP). If a patient is insured with GHP (~ 30% of Geisinger patients), information for each patient claim with the insurance company and details surrounding that claim are available, thus capturing claims that may occur outside of Geisinger facilities
Brain Imaging	Access to brain imaging (including MRI and CT) is possible using the Geisinger EHR. This clinical data available includes the reason for the ordered imaging, date of diagnosis related to the imaging, findings, etc. Brain imaging information can be linked to the other health record information for each patient generate a more comprehensive health data overview for these patients

#### Self-report questionnaires

As described in the Study Design section above, we capture several self-report questionnaires from enrolled patients as a quantitative estimate of several traits. See Table 1 for complete descriptions and details and Additional File 1 for survey questions that are not part of standardized assessments.

#### Electronic health record (EHR) data

A variety of data types are available within a patient’s EHR. As part of the consent process, patients agree to allow access to their health record for the duration of study enrollment. This allows for completion of chart review on all available data from years before the patient enrolls, as well as longitudinal chart review and data export for months and years following the enrollment date. For the types of information that are available in EHRs, please see more comprehensive reviews [23, 29]. Although the entire EHR is available for the current study, we describe the most relevant variables that are captured in the Geisinger EHR, and focus on any Geisinger-specific programs and data resources. See Table 2 for complete description and details regarding EHR variables.

### Data analysis

We will conduct statistical analyses of phenotype, genetic, and neuroimaging data (both as separate and integrated datasets?). These will include Genome-, Phenome-, and Exome-Wide Analyses to discover genes and clinical phenotypes associated with OUD risk. Analyses will also include regression and multivariate analyses to assess differences in brain structure over a continuum of opioid abuse risk, as determined by a quantitative PO addiction score (see Chart Review, below). Statistical analyses will also combine all of the measures below in an effort to identify a comprehensive and reliable set of risk factors for OUD.

1. *Chart Review.* Enrolled patients’ entire EHR will be reviewed using a rubric-based procedure [11] to determine a quantitative DSM-based OUD severity score.
2. *EHR data analysis.* EHR data from discrete fields will be exported from all enrolled patients’ charts. Common comorbid diagnoses will be determined using cluster and correlation analyses. Patient groups and or case/control status may also be determined using ICD codes for OUD.
3. *Patient-reported questionnaire data.* Patient-reported questionnaire data will be scored according to standard procedures for each assessment. Summary scores will be compared between various groups (e.g. those with and without an OUD diagnosis) and used as covariates in genomic and brain imaging analyses, described below.
4. *Genomic analyses.* Various genetic analyses of data will be performed to identify specific DNA sequence changes that are associated with OUD. Methodologies related to DNA sample preparation, sequencing, sequence alignment, variant identification, genotype assignment, and quality control (QC) steps will be carried out as described in Dewey et al. [28]. For Illumina HOEE genotyping data, SNPs will be called using standard methods in Illumina GenomeStudio. For genotype imputation, genotypes from the HOEE genotyping array will be imputed using the University of Michigan human imputation server (https://imputationserver.sph.umich.edu/). Imputed data will be cleaned using standard QC methods.

#### GWAS analyses

GWAS will be performed by running genotyped, imputed, or WES variants (MAF>1%) against quantitative measures of OUD. The primary analysis will use a mixed linear model to assess the relationship between OUD severity score and SNPs coded additively with respect to the number of minor alleles. Models will be controlled for any ancestry differences using principal components (PCs). To understand possible confounds, covariates, including biological sex, BMI, age, FTND score, AUDIT score, PHQ-9 score, and GAD-7 score will be included in models. Functional enrichment analysis and mapping regulatory variation will be followed up using eQTL approaches using publicly available genet expression databases.

#### Polygenic risk score (PRS)

An important benefit of GWAS is to predict the relative genetic risk that individuals may have to develop a particular disease. Knowledge of this risk can then be used for prevention, diagnosis, prognosis, and treatment of a disease. To estimate the genetic liability for OUD in individual patients, we will use a polygenic risk score (PRS) approach, summarizing the genetic effects among an ensemble of markers across the genome.

#### Rare variant analyses

Although GWAS will identify common variants (MAF > 1%) contributing to OUD risk, it is important to consider how rare coding (or even non-coding) variants and/or CNVs (MAF < 1%) within genes lying closest to GWAS hits may contribute to OUD risk. Rare SNPs within the same gene will be binned and then analyzed using a variety of aggregate techniques, including burden tests and kernel-based association methods (e.g., SKAT; [30]).

#### Brain imaging analyses

Available MRI data are extracted from the clinical picture archiving system and de-identified through a Geisinger data broker using existing Geisinger protocols. Briefly, patients with available MRI data are identified based on corresponding CPT codes for MRI of the head/neck. Image accession numbers are de-identified and corresponding image headers stripped of protected health information. Images are uploaded to a research picture archiving system and linked with corresponding patient data using study IDs. Images are run through a quality control process and gray matter, white matter, and cerebrospinal fluid volumes extracted using commonly used and available neuroimaging software. Output volumes can be used in a series of analyses relating brain volumetrics to phenotypic and genomic data.

## Discussion

The study design described here demonstrates the utility of harnessing several real-world clinical resources and the translational research infrastructure within an integrated health system, Geisinger, for the purposes of scientific discovery. The primary goal of our current work is to use these resources to discover novel risk genes associated with OUD, but this study design can also be useful as a model for understanding comorbidities or other complex diseases. The combination of EHR data capture along with patient reported information expands the potential of this data source for deep, high-throughput phenotyping, enabling the identification of thousands of patients with well-characterized opioid use history in a relatively short time frame.

Previous research in OUD predominantly focused on patients who used illicit opioids and are in a treatment setting. Many intermediate opioid phenotypes are lost when opioid use behaviors are condensed to define individuals as either cases or controls. EHR data offer the opportunity to better understand the full continuum of opioid phenotypes, ranging from exposure to addiction. The integrated nature of Geisinger’s health system, including embedding addiction medicine clinics into a whole-patient treatment model, also allows for an unique opportunity to understand the spectrum of opioid using patient phenotypes, including those in the context of chronic pain treatment, as well as those in an active treatment setting with a confirmed diagnosis.

One of the primary goals of this effort is to capture deep phenotyping for use in genetic analyses. Although diagnostic codes from EHRs have been used extensively for genetic discovery of many medical diseases [15–17], the use of EHR data for case/control definition of psychiatric disease is still evolving. One challenge regarding patient characterization within this study is that of phenotype stability and case–control status. Previous GWAS of OUD used a range of control definitions, including family-based characterization involving interviews [12], populations characterized by opioid misuse [31]; as well as definitions that require at least one documented opioid prescription [14]. OUD case characterization is a less daunting challenge; OUD is a lifetime diagnosis, so every individual with an OUD treatment history can be considered a true case. The real challenge will be validation of OUD diagnoses in patients not in treatment. These patients may lack an OUD diagnosis in the EHR, based on ICD codes and/or treatment at a substance use disorder clinic. Previous epidemiological work within Geisinger has demonstrated that many people have OUD based on chart review and/or patient interview, even when that diagnosis has not been formally conferred [10, 11]. Patients with pre-existing nicotine use disorder, anxiety, and/or mood disorders tend to have greater numbers of OUD symptoms following opioid exposure and/or treatment of non-progressive pain with POs [6, 7], for review, see [32]. Thus, when a genomic analysis is completed, there may be patients with opioid exposure that have not yet developed OUD, but due to other factors, are at particularly high risk. Given the complexities of defining such opioid-using ‘controls’, the use of more sophisticated multivariate modeling, such as Genomic structural equation modeling [33] that can account for these comorbidities and risk factors may be best for uncovering genetic differences associated with OUD.

MRI analysis has contributed substantially to our understanding of brain development, aging, and cognitive processing. Studies of brain structure that examine group-level differences tend to utilize prospective research recruitment strategies, requiring substantial time and money to assemble large population cohorts. Conversely, health care systems amass large collections of MRIs as part of routine patient care, but clinically-ascertained imaging tends to only be used for small cohort or case studies. To our knowledge, this study represents the first to extract thousands of brain MRIs from the EHR and use these to better understand opioid use and OUD. Although MRIs from clinical care are limited to brain structure measurements, there have been previous findings of altered brain structure from chronic pain [34] as well as use of opioids [18, 19]. There is also a body of work indicating evidence for structure–function relationships in the brain (for reviews, see [35, 36]), including our own work examining structural markers in the orbitofrontal cortex [37, 38], a brain region thought to be associated with risk for substance use disorders [39–41], Individual MRI metrics, such as a measurement of brain volume in a given region of cortex, can then be integrated statistically with phenotype and genetic data to evaluate the involvement of specific neuroanatomy in mediating relationships between genotype and phenotype (for review of approaches, see [42]).

The Geisinger health system translational research infrastructure is unique in many ways, contributing to the potential of the current study. Namely, Geisinger is one of a few integrated health systems in the United States, serving a largely rural population with a very low outmigration rate. Geisinger is also the primary health system serving patients within its geographical service area. Thus, there is incredibly dense data capture for large portions of a given patient’s lifetime. With Geisinger’s health insurance plan and integration of national prescribing data, a complete picture of a patient’s health and treatment course can be captured from existing resources. Other US health systems with large biobanks (e.g. Vanderbilt University Medical Center’s BioVU (https://www.vumc.org/dbmi/biovu), Massachusetts General Brigham Biobank (https://biobank.massgeneralbrigham.org)) are located in urban geographical regions and treat patients from a more diverse and geographically wide patient base. Certainly, other health system biobanks offer other distinct and meaningful data capture that is not present in the Geisinger population (e.g. more racially diverse populations, see Limitations, below). In addition to the distinct features of Geisinger, there are challenges across all biobanks with shifting clinical diagnosis and prescribing trends in all U.S. health systems, given ongoing efforts to limit opioid prescribing and increased recognition, identification, and treatment of patients with OUD [43–45].

There are several limitations of the type of data captured as part of this study. This sample will be limited to those that use the health care system. In addition, our patients that are identified as having OUD within the context of addiction medicine treatment will be composed of treatment-seeking patients, which is a small subsample of patients with OUD [46]. Although our recruitment sample is quite large (currently 10,000 people), this sample size is still very small relative to the estimated size needed for well-powered GWAS analyses. For this reason, and based on the population characteristics at Geisinger, we prioritized patients with European American ancestry to maximize statistical power. Prescription OUD and the ongoing epidemic stemming from prescription opioid abuse is also more prevalent in European American populations. Given the limited racial diversity of our proposed sample, we aim to participate in multi-site endeavors that are outside of the immediate scope of this funded work to better understand this phenotype and any genetic findings in more diverse populations.

Another limitation exists in the challenges of EHR phenotyping and cohort effects that are present in all health system biobanks. One salient example is our own observation that diagnostic practices surrounding OUD diagnoses have dramatically changed over the course of the past few years at Geisinger. As recently as 5 years ago, the stigma and lack of physician education surrounding OUD stemming from Pos given during routine clinical care resulted in very few individuals being diagnosed. There were several Geisinger system-wide initiatives to reduce stigma, increase patient identification, and reduce opioid prescribing that may have accelerated changes in the health system. Further, one could argue that the most severe disease exists in patients who never present for treatment, either because the treatments as currently available require patient compliance and participation, or because a number of the most ill will die before entering treatment. Efforts and associated funding to reach and characterize the most severely impacted of the OUD spectrum is a pressing need for future research.

Here, we describe a study that recruits a large patient population using a combination of research and clinical infrastructure within one integrated health system, Geisinger. With this study, we aim to provide a platform for clinical and genetic discovery related to opioid use and abuse. To capture similar information within populations using other health system biobanks, it will be critical to close existing gaps in relevant data capture. We and others have shown that PO data are valuable for identifying patients at high risk for developing OUD [11, 14, 47–50]. However, these studies can only be completed in distinct populations where prescription drug histories are captured with relatively high density. For example, outside of the current study, other work has used cohorts such as military veterans, who receive all of their care within the same infrastructure [14, 50] or have drawn from large, population-based cohorts of medically insured adults [48]. Prescription drug information is captured at the state level, with most states maintaining a prescription drug monitoring program for clinical use. National programs also exist; for example, Surescripts maintains an e-prescribing database that captures dispensed drug information from most retail pharmacies across the United States. To facilitate identification of individuals who have a high likelihood of being OUD cases, the approved use of state-wide and national prescription drug monitoring programs for research purposes is a necessary step that will enable scientific discovery and dramatically improve patient identification and treatment.

## Supplementary Information

**Additional file 1**. Instructions and Survey Questions for DSM self-report scale (e.g. Fig. 1). Questions and scoring procedure for adapting DSM questions for opioid use disorder into a self-report format.

## Data Availability

At the end of the study, data captured as part of **GaPO** will be deidentified and deposited in public repositories. Data requests can also be made by contacting the corresponding author, Vanessa Troiani, by email: vtroiani@geisinger.edu.

## Abbreviations

GaPO: Genetics and Prescription Opioid
OUD: Opioid Use Disorder
SNP: Single Nucleotide Polymorphism
GWAS: Genome Wide Association Study
EHR: Electronic Health Record
MRI: Magnetic Resonance Imaging
BRQ: Brief Risk Questionnaire
FTND: Fagerstrom Test of Nicotine Dependence
AUDIT: Alcohol Use Disorders Identification Test
GAD-7: Generalized Anxiety Disorder
PHQ-9: Patient Health Questionnaire
DSM: Diagnostic and Statistical Manual of Mental Disorders
PRS: Polygenic Risk Score
