# Supplementary material for: Genetics and prescription opioid use (GaPO): study design for consenting a cohort from an existing biobank to identify clinical and genetic factors influencing prescription opioid use and abuse
Source: BMC Med Genomics. 2021 Oct 26;14:253. doi: 10.1186/s12920-021-01100-z (PMC8547564; doi:10.1186/s12920-021-01100-z)
Supplement: Supplementary file 1 — Additional file 1. Instructions and Survey Questions for DSM self-report scale (e.g. Fig. 1). Questions and scoring procedure for adapting DSM questions for opioid use disorder into a self-report format. [file 12920_2021_1100_MOESM1_ESM.docx]

**Supplemental Text:**

Instructions and survey questions used to determine DSM criteria for opioid use disorder:

*Instructions for participant:* Please answer the following questions with reference to the time that your opioid use was the greatest.

All questions have yes or no options.

1. When you start to use opioids, do you often use more than you intended or use for longer periods?

2. Have you made frequent attempts to reduce your use of opioids, but couldn't?

3. Have you spent a lot of time using opioids or doing whatever you had to do to get opioids?

4. Have you had strong urges to use an opioid?

5. Have you ever missed work, school, or other responsibilities because you were very high or recovering from opioid use?

6. Has your opioid use caused arguments or fights with other people, including family members, friends, or people at work?

7. Would you often use opioids instead of spending time with friends/family or going to work?

8. Have you ever used an opioid when it might have been dangerous, such as driving a car while high?

9. Did you continue to use opioids even though use caused psychological or physical problems in your life?

10. Have you taken larger amounts of opioids to get the same effect?

11. Have you found that your usual opioid dose is less effective?

12. Have you ever suffered from anxiety, sweating, restlessness or insomnia when opioids were not available?

13. Did you ever take an opioid or similar drug to avoid withdrawal?

14. Did you ever have trouble with the law because of opioid use?

Each item is scored as yes or no in response to whether the statement describes the participant’s drug use. Yes responses are scored as a 1 and no responses scored as a 0. Questions 1-9 are scored as 0 or 1. For answers to questions 10 and 11, which both correspond to Tolerance criterion, a positive answer for either item results in a 1 for the Tolerance criterion. For answers to questions 12 and 13, an endorsement of either question results in a score of 1 for the Withdrawal criterion. Responses are then summed for a total score. In order to adapt summary scores to different DSM criteria, questions relevant to a specific criterion can be included or ignored in the summary scores to compute a DSM-specific score. For example, abuse and dependence differentiation utilized in DSM-IV can be calculated by summing answers to questions 5, 6, 8, and 14 for abuse and questions 1,2,3,7,9,10,11,12, and 13 for dependence. Legal trouble is not included in DSM-5 criteria, so question 14 is excluded to calculate DSM-5 scores using questions 1-13.
